# LUCIA: An open source device for disinfection of N95 masks using UV-C radiation

**DOI:** 10.1016/j.ohx.2021.e00181

**Published:** 2021-02-26

**Authors:** Marcel Bentancor, Sebastián Fernández, Federico Viera, Sarita Etcheverry, Carolina Poradosú, Pablo D'Angelo, Hernán Montemuiño, Santiago Mirazo, Álvaro Irigoyen, Analía Sanabria, Horacio Failache

**Affiliations:** a Laboratorio de Biología Molecular Vegetal, Instituto de Química Biológica e Instituto de Biología, Facultad de Ciencias, Universidad de la República, Iguá 4225, 11400 Montevideo, Uruguay; b Instituto de Ingeniería Eléctrica, Facultad de Ingeniería, Universidad de la República, J. Herrera y Reissig 565, 11300 Montevideo, Uruguay; c Escuela Universitaria Centro de Diseño, Facultad de Arquitectura, Diseño y Urbanismo, Universidad de la República, Juan D. Jackson 1325, 11200 Montevideo, Uruguay; d Sección Virología, Instituto de Química Biológica, Facultad de Ciencias, Universidad de la República, Iguá 4225, 11400 Montevideo, Uruguay; e Linebay S.A., Buenos Aires 618, 11000 Montevideo, Uruguay; f División Laboratorio Ambiental, Dirección Nacional de Medio Ambiente, Ministerio de Ambiente Av., Italia 6201, 11400 Montevideo, Uruguay; g Instituto de Física, Facultad de Ingeniería, Universidad de la República, J. Herrera y Reissig 565, 11300 Montevideo, Uruguay

**Keywords:** Virucide, Pandemic, SARS-CoV-2, COVID19

## Abstract

Faced with a global pandemic such as the one triggered by the SARS-CoV-2 virus, the medical supply chain has been highly demanded. An item in which this manifested itself more clearly, are the N95 masks, designed to be disposable items, in many cases they have had to be reused. In these emergency conditions, it was necessary to apply an effective and safe method that can be used locally. Here a device for disinfection by ultraviolet C light was developed that allows irradiating N95 masks with a known and reproducible dose. Thus being able to apply a safe and effective disinfection method according to existing information. The use of a common model of UV-C lamps and the simple construction of the device allows it to be built at low cost and with widely available materials. The effectiveness of the device was demonstrated against an enveloped RNA virus, characteristics shared with the virus that causes COVID19, being capable of reducing the viral load by 4 orders of magnitude.

## Graphical abstract

**Figure:**
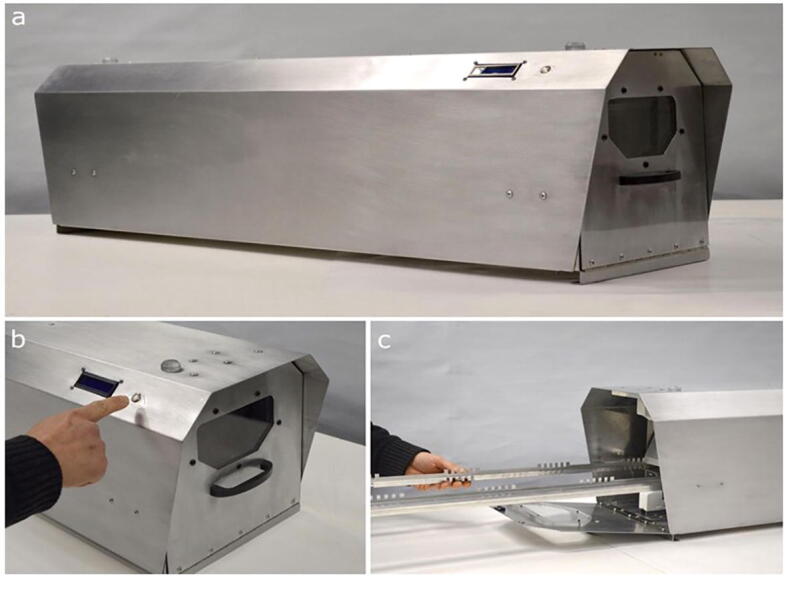

**Specifications table**

**Table:** 

Hardware name	LUCIA: Disinfection device for N95 masks.
Subject area	• Medical • Biological Sciences
Hardware type	• *Other [disinfection device]*
Open Source License	*CC 4.0*
Cost of Hardware	*U$S 360,00*
Source File Repository	https://doi.org/10.17632/cpzfdw95ty.2

## 1 Hardware in context

The N95 masks are articles designated to be disposable elements, they always should be used in this way when there is a normal supply of them. For this reason, almost no commercial devices exist as the one developed here. However, in emergencies and when these masks are scarce there is a necessity to extend their use and reuse them [1], [2]. To accomplish this, several strategies have been proposed [3] to allow secure reuse of them for certain additional time. Previously on 2006, the Institute of Medicine (USA), convened a “Committee on the Development of Reusable Facemasks for Use during an Influenza Pandemic” [4]. There it was indicated the necessity to research about methods to decontaminate this kind of mask. Several methods and studies have been reported [5], [6], [7], [8], [9], [10], [11].The three main explored strategies are the application of hydrogen peroxide, dry or wet heat treatment, and exposure to UV-C radiation.

UV-C radiation produces cyclobutane pyrimiden dimers (CPDs) damaging DNA or RNA molecules, so interferes with its replication and expression [12], [13], [14] other mechanisms also could contribute to the biocide effect of the UV-C radiation [15]. The atmosphere blocks almost all the UV-C radiation from the sun, not allowing it to act as a selective pressure, to generate resistance against this radiation. It could be the reason because the UV-C radiation is so effective to destroy pathogens like viruses [16]. This kind of radiation has been used since the XX century as a disinfecting agent and the availability of powerful enough UV-C lamps has allowed the development of open source devices for room disinfection [17]. With this idea in mind, facing the SARS-CoV-2 pandemic and with the need to disinfect N95 masks, protocols for the use of environmental irradiators to disinfect these masks emerged [18]. However, this kind of protocol requires its adaptation for each institution, because it is not frequent to dispose of the same model of UV-C irradiator. It was necessary to have a device that, using UVC lamps widely available in the market and initially intended for other uses (i.e. as spare pieces for laminar flow bench) would allow irradiation with a suitable dose, securely and effectively to N95 masks. We have chosen a model of low-pressure mercury which is widely available on the market, it emits at near 254 nm and is effective as a virucide [19], [20], [21]. It was also sought that the device was of economic construction, and that its operation was simple. The device here presented also has a UV-C meter to automatically adapt the exposure time to reach the required UV-C dosage to decontaminate the N95 masks. The idea to develop UV-C equipment to disinfect N95 masks also was explored by other groups although with different grades of documentation [3], [22]. In this article design files are provided to build and industrialize our device, also a wide characterization of the device’s performance is provided.

## 2 Hardware description

In this equipment we have chosen to use discharge tubes as the light source because they are broadly distributed and are easily affordable. This is an important consideration in countries where these components are imported and not-easily accessible on time due to the present distorted international market. Second, discharge tubes are not expensive in relation to their high power UV-C light emission and is a mature technology probed over many decades.

**Fig. 1:**
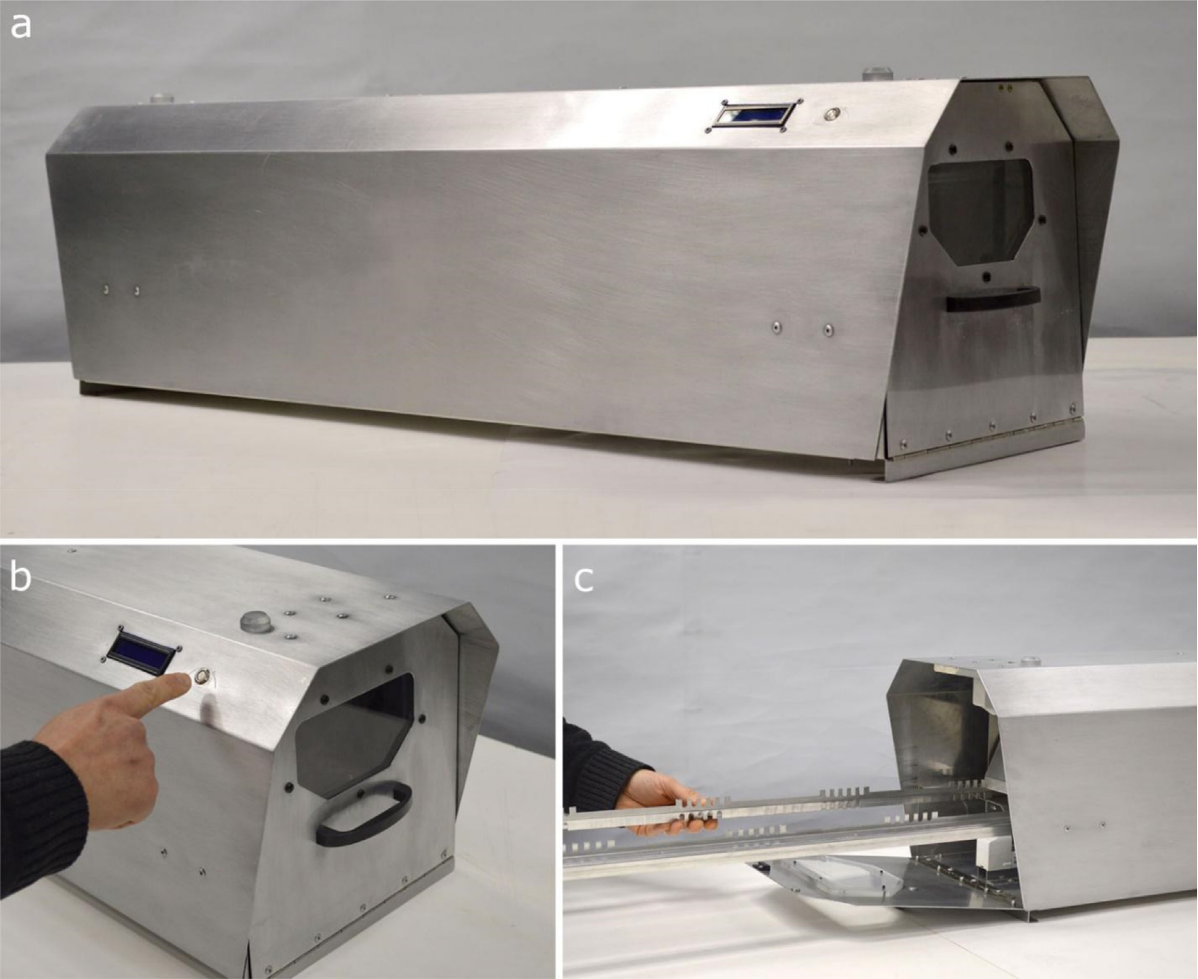
a) General view of the device, it has two doors for input and output of the masks’ support with the N95 masks. Over each extreme there is a led indicator to show the state of the disinfection process. b) Control panel of the device. For an easy operation it only has a button and a LCD display. c) Input of the masks’ support.

To have a proper mask disinfection the UV-C energy must be evenly distributed over the N95 mask surface, both external and internal, avoiding then to under-irradiate any spot on the surface. Special care should be therefore taken to illuminate the irregular mask surface with a light irradiance evenly distributed over it avoiding any shade. We have then created an irradiation chamber with reflecting surfaces, which reduces the number of discharge tubes to be used. In this equipment (Fig. 1) the optical design of its interior is then essential.

The equipment uses three 30 Watt discharge tubes emitting UV light in the C band. These tubes are specially produced for germicidal applications. As described in section 7, they have most of their light emission concentrated in the narrow mercury (Hg) line at 253.7 nm.

The equipment uses an aluminum reflector, that efficiently reflects UV-C radiation, specifically designed to obtain an adequate homogeneity of the radiant energy incident on the surface of the masks, both on their inside and outside, as well as on their straps.

**Fig. 2:**
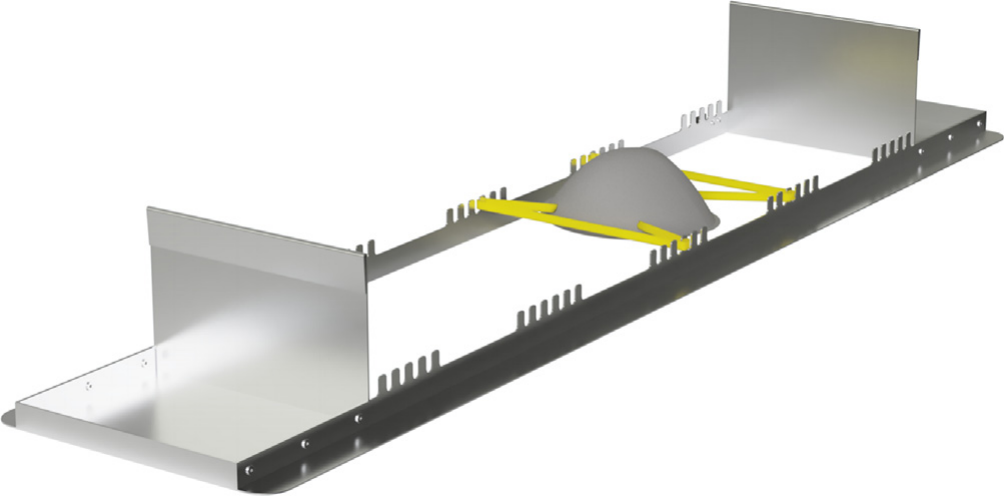
The masks support showing a N95 mask hold by their elastic bands in order to minimize shadows on their surface.

The structure supporting the masks was designed to minimize the shadows on the masks or on their straps. The masks stand in their positions inside the irradiation chamber hold by their straps, as shown in Fig. 2. The attaching points are defined at the edges of the support. Only a maximum of 4 masks is allowed in order to avoid the shadow of one mask to the neighboring ones.

The equipment also incorporates an UV-C sensor, which exclusively detects this germicidal radiation, and which is used to calibrate the dose to be delivered. When the equipment is turned on, it performs a calibration of the radiation emitted by its tubes and which is in turn reflected by the reflectors. This takes into account the effect of aging of the emitter tubes and dust or other types of dirt that deposited on the reflectors can reduce the UV radiation that reaches the masks. This safety device substantially increases the useful life of the equipment while reducing the frequency of maintenance, and reduces the risk of under-irradiation. Moreover, to reduce the risk of an inadequate disinfection, the equipment is also constantly measuring the UV light power inside the irradiation chamber. If a reduction in the UV-C light power is detected, the equipment will automatically turn off and warn the user about a malfunction.

The user interface is simple, consisting of a two-row display used to show the level of radiation applied, remaining doses apply time and notification messages (door open, unload equipment, etc), one visual signal (red and green led) duplicated near each door, one auditive signal (internal buzzer) and a press button. Magnetic sensors are located on the doors and the sliding mask support.

The software is structured in two applications: uv_meter_setup and uv_meter. The uv_meter_setup application is used while assembling and the initial setup of the unit. It can be controlled by sending commands using serial communication. It enables testing the sensor, controlling the notification systems and loads, and stores initial parameters in the EEPROM memory of the microcontroller. uv_meter application is the piece of software used for normal operation. It controls the UV-C source and supervises radiation levels if sensor usage is enabled using uv_meter_setup.

The fact that our group has managed to transfer this design to industry for its manufacture shows that it has passed the prototype stage and that the information and files attached to this article allow its industrialization, being able to manufacture them at low cost. The design and manufacture of this device represents a successful case of industrial transfer of open source technology, in which all parties have benefited: the end-user, academic developers, and the manufacturing industry.

The hardware here described could be useful for researchers outside the original proposed use of this device:

• This equipment takes advantage of commercial UV-C lamps, widely available in the market and that could be in stock as spare parts for other purposes, for example in hospitals or laboratories.
• The construction of a low-cost UV-C radiation meter is included. With minor adaptations, this equipment could be used for the disinfection of other items, different from N95 masks.

## 3 Design files

Design Files Summary

**Table:** 

Design file name	File type	Open source license	Location of the file
*Design file 1*	*ZIP file*	*Creative Commons Attribution-ShareAlike 4.0 International License.*	https://doi.org/10.17632/cpzfdw95ty.2#file-f26ebc6f-3356-4f59-8f0b-74ef74bc67d9
*uv_meter_arduino.ino*	INO file	*Creative Commons Attribution-ShareAlike 4.0 International License.*	https://doi.org/10.17632/cpzfdw95ty.2#file-baebd884-1acd-4a50-8994-2258477735f8
*uv_meter_setup_arduino.ino*	INO file	*Creative Commons Attribution-ShareAlike 4.0 International License.*	https://doi.org/10.17632/cpzfdw95ty.2#file-73eaf381-cd50-4b53-96ff-b55a80649455
*Design file 4*	ZIP file	*Creative Commons Attribution-ShareAlike 4.0 International License.*	https://doi.org/10.17632/cpzfdw95ty.2#file-1582d96d-9320-468f-90ee-d0b648826f0c
*N95_UVC_disinfection_device_CAD.pdf*	PDF file	*Creative Commons Attribution-ShareAlike 4.0 International License.*	https://doi.org/10.17632/cpzfdw95ty.2#file-b1a619b8-84c5-4df5-8405-ee635c952a70

Design file 1, is a ZIP file which contains UVC_METER and UVC_SENSOR circuits design files: Kicad project (schematic and board) and a detailed bill of materials in ODS files.

uv_meter_arduino.ino, is an INO file which has the Arduino code for the software UV_METER.

uv_meter_setup_arduino.ino, is an INO file with the Arduino code for the software UV_METER_SETUP.

Design file 4, is a ZIP file containing the ODG and PDF files describing the flowchart of the UV_METER software.

N95_UVC_disinfection_device_CAD.pdf, is a PDF file containing the CAD diagrams of the device.

## 4 Bill of materials

**Table:** 

Designator	Component	Number	Cost per unit -currency	Total cost-currency	Source of materials	Material type
UV-C tubes	Phillips TUV T8 1 SL/25 UV discharge tubes	3	40	120	Local store	Quartz
Support UV-C tubes	Electronic ballast for discharge tubes + support	3	7	21	Local store	Metal, Semiconductor
–	Magnets to hold in place the support of masks and the doors.	3	1	3	Local store	Metal
–	Mounting materials (screws, rivets, etc.)	–	–	10	Local store	Metal
–	Wiring materials (wires, isolating tape, glue, etc.)	–	–	10	Local store	Metal, polymer
Windows	Acrylic polycarbonate compact (20 cm × 12 cm)	2	4	8	Local store	Polymer
DC power	DC power source 9 V, 1 W 110–220 VAC input	1	8	8	Local store	Semiconductor, polymer
Aluminium	Building material for the device case (sheet: 1.0 m^2^, width: 1.25 mm)	2	40	80	Local store	Metal
PCB Boards	Microcontoller board and UVC sensor	1	5	5	jlcpcb	FR4 + Copper
U1	Op. Amp MCP6241T	1	1	0.29	**[a]**	semiconductor
D1	UVC photodiode GUVC-S10GD	1	9	9	**[b]**	semiconductor
A1	Arduino Nano	1	15	15	**[c]**	Other
2 row display	2 Row display with I2C interface	1	9	9	Local store	Other
K1	5v relay	1	1.5	1.5	Local store	Other
Plastic case for uvc meter	19 cm × 4 cm × 8 cm	1	9	9	Local store	Plastic
Led red/green	5 mm bi-color led	2	0.6	1.2	Local store	semiconductor
Magnetic sensors	Normally Open magnetic sensor	3	7.7	23.1	Local store	Other
Push button	Normally Open metallic push button	1	3.9	3.9	Local store	Other
W*	Wire Terminals 2 and 3 contacts	5	2.6	13	Local store	Other
R*	Various resistors	1	3	3	Local store	Other
C*	Various Capacitors	1	5	5	Local store	Other

**[a]**https://www.digikey.com/product-detail/en/microchip-technology/MCP6241T-E-OT/MCP6241T-E-OTCT-ND/1979750.

**[b]**https://www.digikey.com/product-detail/en/genicom-co-ltd/GUVC-S10GD/2096-GUVC-S10GDCT-ND/10475469.

**[c]**https://www.digikey.com/product-detail/en/arduino/ABX00033/1050-ABX00033-ND/10239972.

Detailed bill of materials of circuit boards component is included in the design files along with the schematics and board.

## 5 Build instructions

In the following instructions we assume that you have previously constructed all mechanical parts described in the corresponding CAD file and the electronics circuitry were built and you have all the required parts as magnetic sensors, display, button, led lights, main plug, switch, cables, etc.

**Fig. 3:**
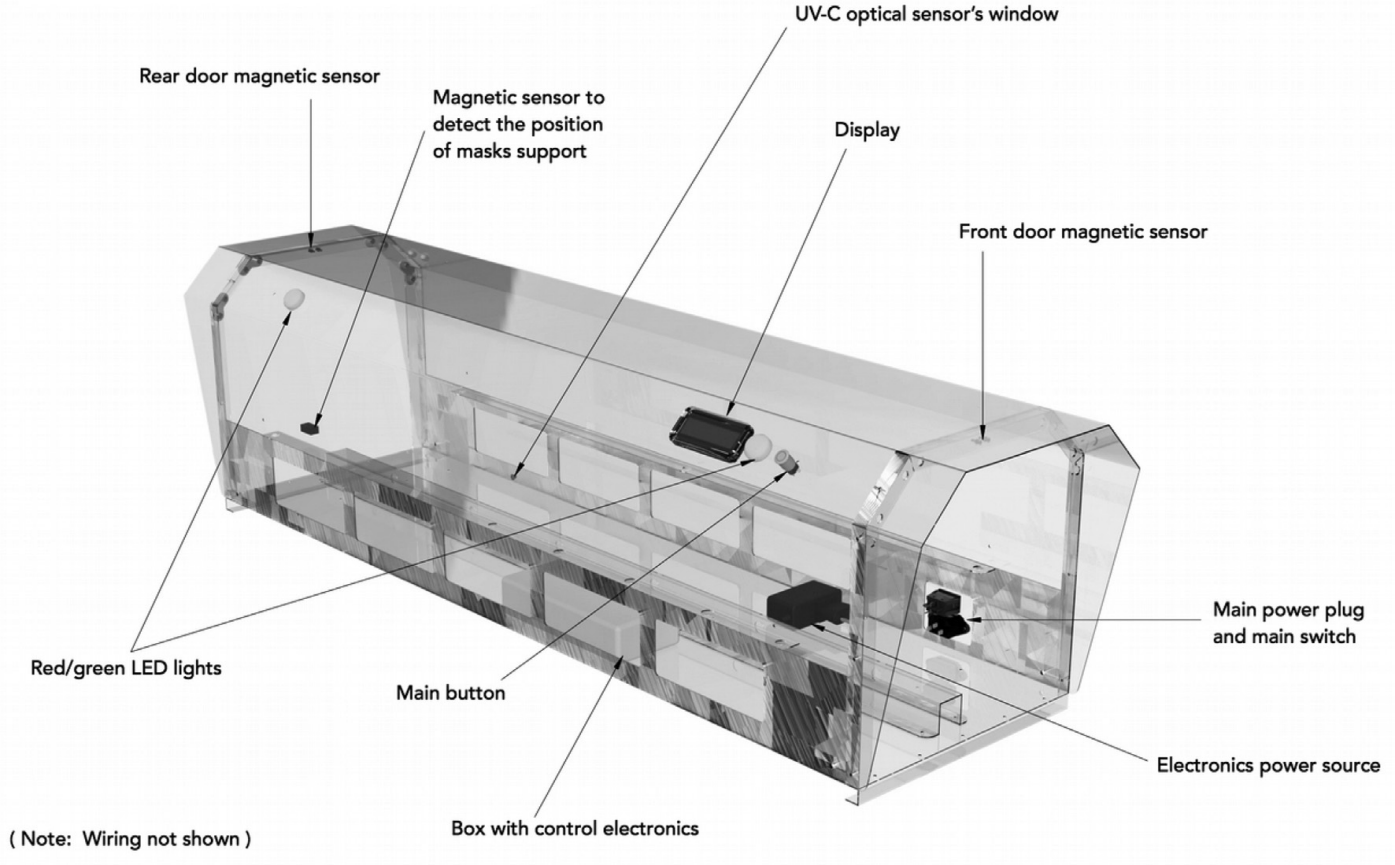
Placement of the different electrical and electronic components.

**Fig. 4:**
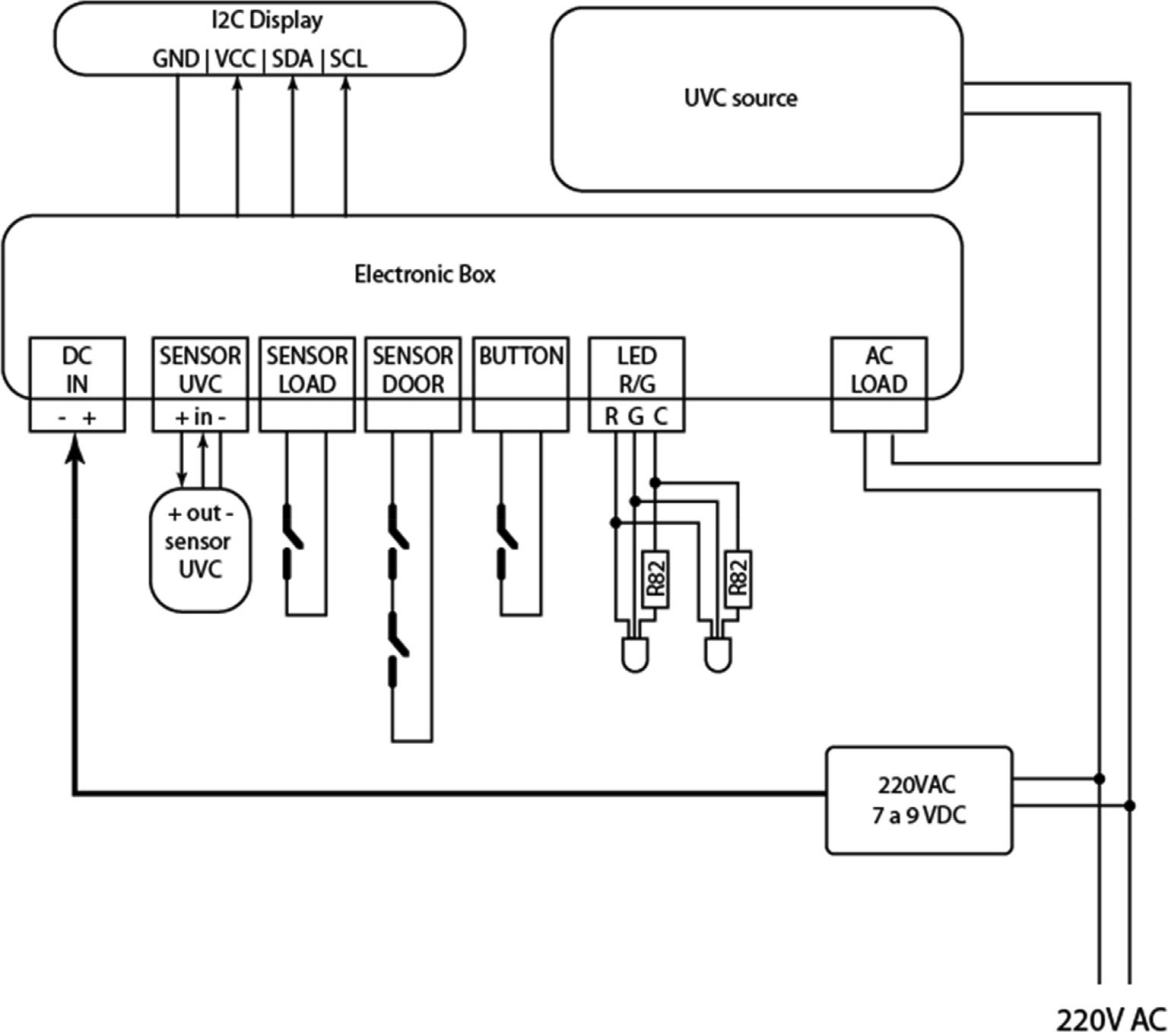
Wiring diagram. UVC lamps are generically indicated as UVC source. Relevant connections are labeled.

Step by step instructions:

1. Before building the device, become familiar with the pieces and how they are assembled together. This is described in the sheets 4 and 11 of the mechanics CAD file. A general view of the main components is shown in Fig. 3.
2. Start by fixing the “W” joist (CAD sheet 15) to the “U” bottom case (CAD sheets 12–14) using rivets.
3. Fix the M6 nuts shown in sheet 12 to the “U” bottom case.
4. Fix using rivets the doors hinge (CAD sheet 16) to the “U” bottom case (CAD sheets 12,14).
5. Screw the lower UV-C tube support (CAD sheets 22–23), including the ballast electronics, to the “W” joist (CAD sheet 15).
6. Fix the main power plug and switch to the “U” bottom case (CAD sheets 1–2,4,12,14, 30). Wire them carefully isolating any exposed conductor.
7. Fix the electronics power source to the “U” bottom case (CAD sheet 30) and connect the source to the main power entrance as described in the wiring instructions (Fig. 4).
8. Connect the lower UV-C tube as described in the wiring instructions.
9. Fix the box with the control electronics to the “U” bottom case (CAD sheet 30) and connect the power source as described in the wiring instructions. Be sure to have access to the electronics terminals through the inspection windows of the “U” bottom case (CAD sheet 12).
10. Fix using rivets the 4 pieces of the bottom cover (CAD sheets 11, 19).
11. Fix both upper tubes to the upper mirror (CAD sheet 22–23).
12. Joint together, using rivets, the lower mirror (CAD sheet 24) and upper mirror (CAD sheet 25) using the “S” profile (CAD sheet 26).
13. Fix using screws the joined mirrors (CAD sheets 22–23) to the “U” bottom case (CAD sheet 11).
14. Wire the upper tubes, lower tube and connect them to the control electronics as described in the wiring instructions (Fig. 4).
15. Plug the power source to the electronics.
16. Fix the UV-C photodetector in the place shown in CAD sheets 25,30. Take special care to clear the photodiode window with organic solvent before fixing the PCB in place. Wire and connect the photodetector to the electronics box, as shown in Fig. 4.
17. Assemble using rivets the masks support (CAD sheet 27). Fix the magnetic sensor.
18. Slide the masks’ support between mirrors and put it in its right position. Fix, on the joined mirrors, the magnetic sensor that determines the position of the masks support. Before fixing the sensor, verify that it is effectively detecting the magnet of the masks’ support. Wire and plug the sensor to the electronic box (Fig. 4).
19. Assemble the top case. Start fixing using rivets the upper and top central cover pieces (sheet 20–21).
20. Assemble the door's frames (sheets 5, 9–10) and fix them to the upper case using rivets.
21. Fix the display, the LED lights, the button, and the doors magnetic sensor (sheet 30). Wire them as described in the wiring instructions. Use long enough cables to plug them to the electronics box before putting in place the upper case.
22. Assemble the doors. Fix using screws the windows, the handles, the magnetic sensor and glue the magnet (sheet 16).
23. Fix the doors to the main device using rivets.
24. Before putting on the upper case, plug to the electronics box the display, the button and the doors magnetic sensors.
25. Load the firmware to the microprocessor.
26. Put in place the upper case.

**Wiring and electronics diagrams**

## 6 Operation instructions

The masks are arranged on a support whose objective is to properly locate them within the irradiation chamber. To avoid all kinds of shadows on the masks, this stand is essentially a frame that holds the masks suspended from their reins. This support allows you to hold up to 4 masks. It is introduced by sliding it into the irradiation chamber by opening a gate located at the “dirty” entrance to the equipment (that should be indicated in red). Once the disinfection process is finished, the support with its masks is extracted by opening another “clean” hatch located at the other end of the irradiation chamber (that should be indicated in green). The irradiation chamber remains closed during disinfection, thus avoiding irradiating the operators. The UV-C emitters only turn on when an operator indicates it by means of a button, and only if the gates are closed. Detectors in the gates detect a premature opening of any of them and turn off the UV-C emission to avoid an operator exposure to this radiation. The process then continues when the doors are closed. High-intensity UV-C radiation, such as that used by this equipment, can have an effect on the filtering capacity and on the mechanical resistance of the constituent materials of the masks. Although this effect is unimportant [23], [24] there is no consensus in the literature on the maximum number of times it is advisable to disinfect a mask. We consider 3 as a conservative number of disinfections [4], [5], [25]. This number of disinfections multiply by 3 the effective number of masks available for use in the current pandemic. Therefore, these must be identified with the name of the user and with the number of disinfections to which it has been subjected. After decontamination, is important that each user verify a well adjust of the mask to his face [26]. At the institutions where the units were installed, a protocol to organize the handling of the masks was made and it should be adapted for every institution [2]. As general guidelines for the management of the masks previous documents could be used [18], [27].

## 7 Validation and characterization

Two validation tests were conducted to characterize the performance of the device. We have characterized the UV-C radiation distribution inside the irradiation chamber and we have also proceeded to make a biological validation to test the effective disinfection capacity of the device.

The biological validation tests were performed on a first version of the prototype that only used two lamps. To decrease the disinfecting time a third lamp was added in a second prototype, which is depicted in the attached CAD files (design file 6).

### 7.1 UV-C characterization

**Fig. 5:**
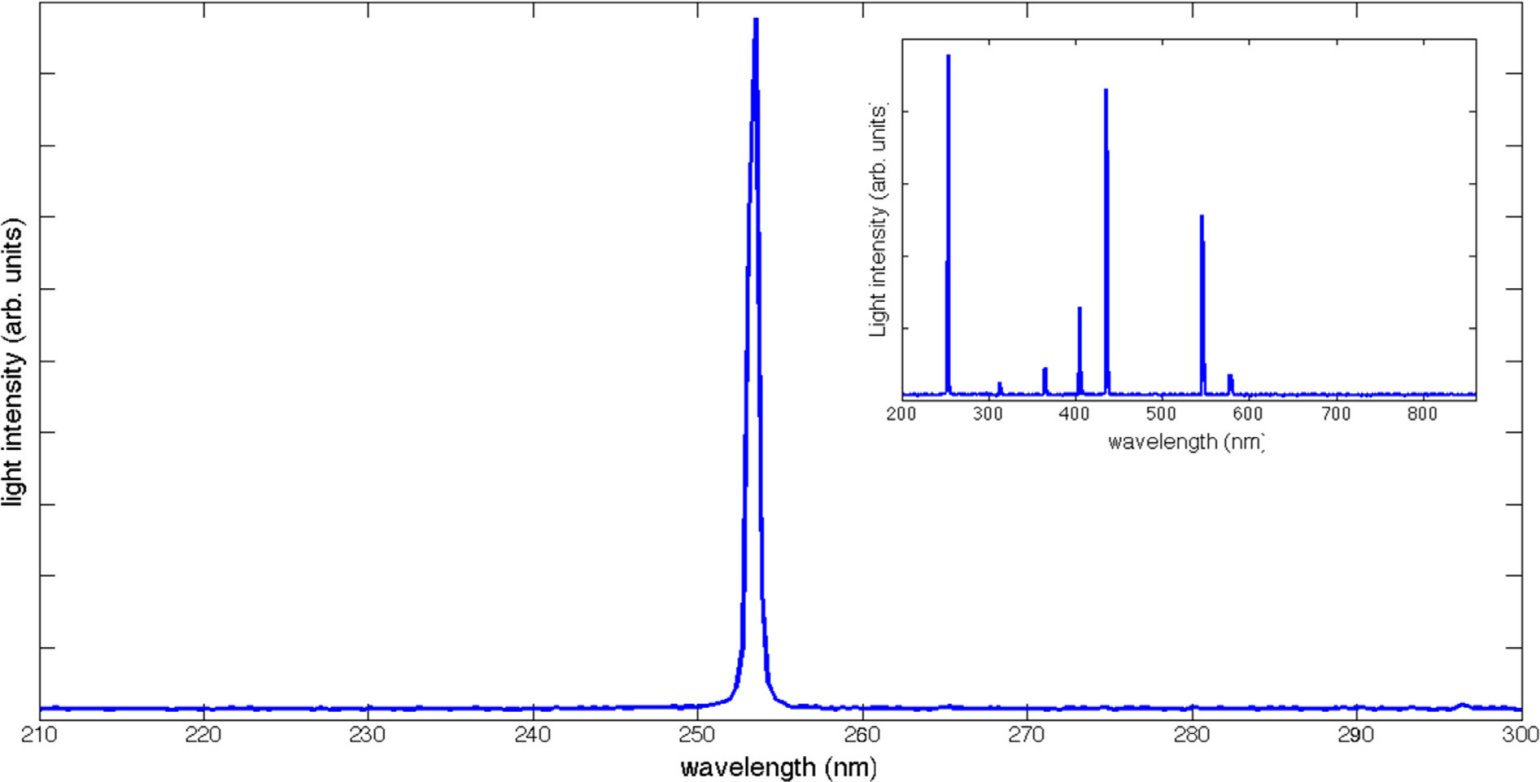
The light emission spectrum of Phillips TUV T8 1 SL/25 tubes in the germicidal band measured using an Ocean Optics S2000 spectrometer. Inset: spectrum including the visible band.

The equipment uses as the UV-C sources a 30 Watt Philip discharge tubes model TUV T8 1 SL / 25 UV. These tubes are specially produced for germicidal applications. As shown in Fig. 5, the emission in the germicidal spectral band (220 – 280 nm) is concentrated in the narrow mercury (Hg) line at 253.7 nm. Moreover, these lamps are ozone free, as they are covered with a filter for the 185 nm wavelength of light, which is the light responsible for the O_3_ production.

We have carefully measured the light intensity in the irradiation chamber, in order to characterize their distribution over the surface of the masks. We have measured some characteristic points distributed inside the irradiation chamber. For each point, we have measured the intensity received on a surface having different orientations, either for the radiation arriving at the external or internal surface of the mask (see Appendix-I for details).

The UV-C radiation delivered by the equipment inside the irradiation chamber was measured with the ZED SMART METER with a calibrated D-SiCONORM sensor (SN:25437). Physically this sensor is relatively big, which makes it difficult to measure every point and orientation inside the irradiation chamber. To make a full characterization of the UV-C intensity (mW/cm2) distribution inside the chamber we have also used the much smaller UV-C photodetector designed and built for the equipment after proper calibration.

To guarantee that every point in the mask's surface receives the minimum required UV-C energy dose, is important to identify the least irradiated points. The minimum intensity received by a point on the surface of a mask was determined to be approximately 2.0 mW/cm2. We have then fixed the exposure time of our equipment based on this value. The recommended dose of UV-C energy is 1–2 J/cm^2^ [16], [20], [21], [22]. A dose of 1 J/cm^2^ is obtained for the least irradiated point in approximately 9 min, considering that when the tubes are turned on they have an initial thermal transient of approximately one minute, in which the intensity increases from a value close to half that of its regime value. A dose of 2 J/cm^2^ is then obtained for approximately 17 min of irradiation time.

### 7.2 Biological validation

A virus titration was assayed to evaluate the disinfecting power of the device. Because SARS-CoV-2 requires an installation with a biosafety level not available in our institution, we used an enveloped RNA virus with similar characteristics. Disinfectant efficacy of UV light device was evaluated with Human Respiratory Syncytial virus (hRSV), Long strain (ATCC VR-26D), *Paramyxoviridae* family, genus Pneumovirinae. HEp-2 cells (ATCC CCL-23) used for viral inoculation were grown in Dulbecco MEM with 10% Fetal Bovine Serum (LifeTechnologies, USA) and 1% v/v antibiotic–antimycotic solution (LifeTechnologies, USA), in a 5% CO_2_ atmosphere at 37 °C. Nitta® N95 face masks Serie 9510 (Colombia) were used for all the procedures.

### 7.3 Disinfection procedure

**Fig. 6:**
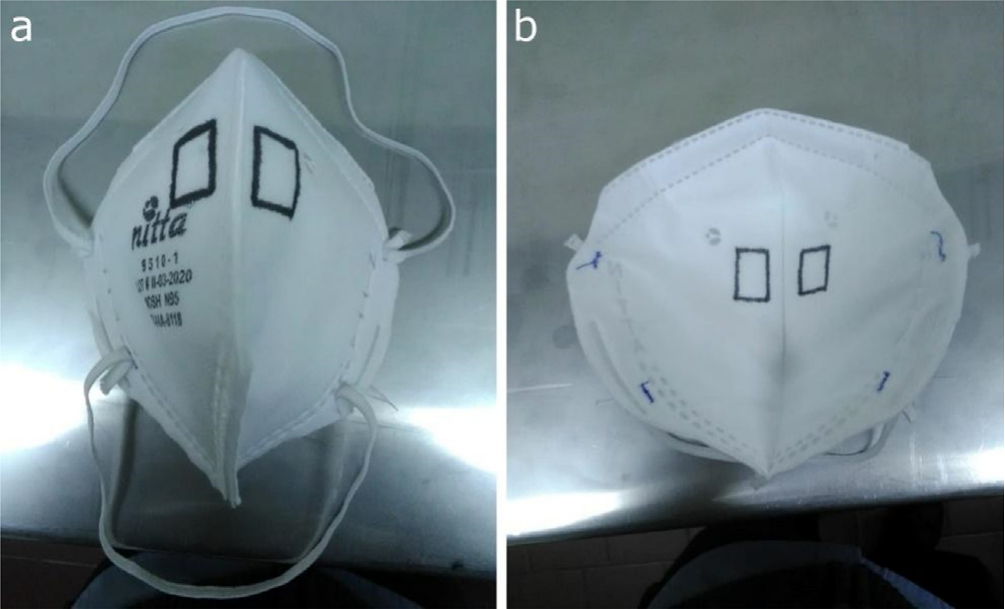
Representative image of locations where the masks were inoculated, outside surface (a) and inner surface (b).

Face masks were inoculated in triplicate at inner and outer sides with 1 ml of a viral suspension containing 5 log_10_ TCID50/ml or 10 log_10_ TCID/ml (Fig. 6). Inoculum was air dried and masks were incubated inside the UV device for 15 min. Experimental conditions were 15 °C and 40% relative humidity. As control of viral survival, equal amounts of inoculated face masks were kept for 15 min under the same conditions but without UV exposition. A total of 24 face masks were employed for the experiments.

### 7.4 Virus titration

After 15 min, inocula from treated and untreated face masks were eluted in 10 ml of phosphate buffer saline (PBS) and 10-fold serial dilutions were performed. Virus titre in each case was calculated by the TCID50 method, as described [28].

### 7.5 Statistical analysis

Differences in the virus titre between treated and untreated face masks and between inner and outer sides were compared using 2-way analysis of variance (ANOVA). A probability of p < 0.05 in this test was considered statistically significant.

### 7.6 Results

**Fig. 7:**
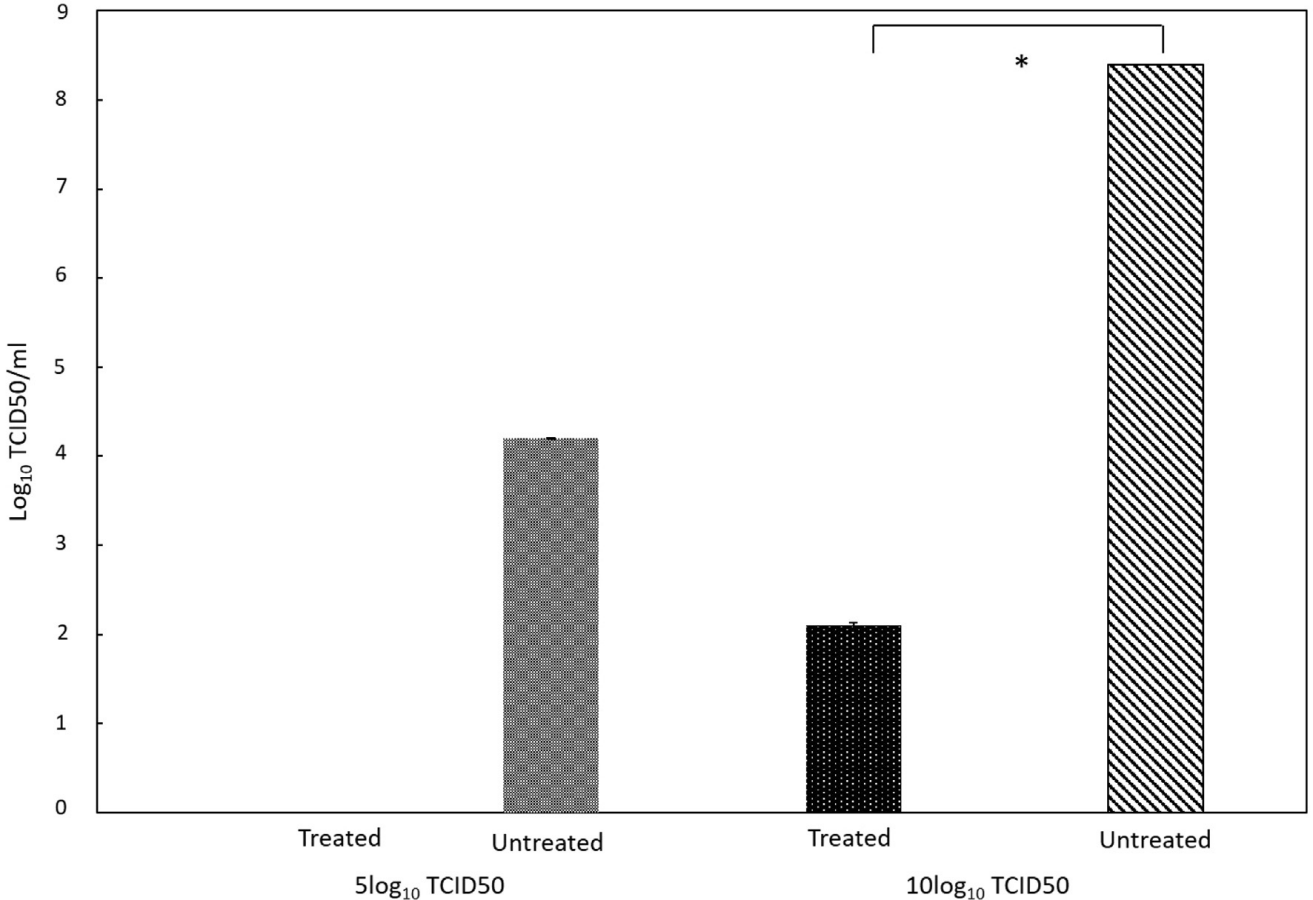
Human Respiratory Syncytial Virus (hRSV) titre reduction by UV exposure after 15 min. Face masks were inoculated with 5 log_10_ and 10 log_10_ TCID50. Inoculated untreated face masks were included as a control of virus survival. Bars show the mean ± SD of three replicates for each condition. SD values were small for all measures and may not be clearly displayed. * p = 0.00005.

After 15 min of UV exposure, infective virus could not be recovered from any of the face masks inoculated with 5 log_10_ TCID50. Mean virus titre of the eluted inoculum from untreated masks was 4.2 ± 0.21 log_10_ TCID50 (Fig. 7). On the other hand, the virus titre eluted from the treated masks inoculated with 10 log_10_ TCID50 was 2.1 ± 0.18 log_10_ TCID50 whereas for the untreated control it was 8.4 ± 0.24 log_10_ TCID50, p = 0.00005 (Fig. 7). No significant difference was observed when the face masks were inoculated in the inner or outer side, with both inocula, p > 0.05 (Table 1).

**Table 1:** Virucidal effect of exposure to UV-C in Human Respiratory Syncytial Virus (hRSV).

Initial viral inoculum* (TCID50)	Exposure time/UV-C dose (mW/cm^2^)	Remanent virus titre/ml after exposure (TCID50). Mean ± SD	Fold-reduction**	p value
5log_10_	15 min/ 2–4	0	>4	
	15 min/ 0	4.2 log_10_ ± 0.21	–	
10log_10_	15 min/ 2–4	2.1 log_10_ ± 0.21	>5	0.00005
	15 min/ 0	8.4 log_10_ ± 0.24	–	

*Three masks were evaluated for each viral dose, at both inner and outer sides (N = 24). No significant differences were observed when the face masks were inoculated in the inner or outer side, with both initial viral doses, p > 0.05.

**Compared to the non-exposed control.

The device here created was able to disinfect N95 masks descending the hRSV viral load, a surrogate virus of the SARS-CoV-2 virus, used for its biological validation. The obtained descending in the viral load is comparable to the case of the SARS-CoV-2 virus, because like other coronaviruses it is particularly susceptible to UV-C radiation [29], [30], [31], [32], [33]. The hRSV here used shares come key points with the SARS-CoV-2 virus: both are single-strand RNA viruses, and they have a lipidic envelope. The reports generated in the last months shown the utility of this kind of light to disinfect materials contaminated with the SARS-CoV-2 virus. The predicted susceptibility of the hRSV to UV-C light is similar to the known susceptibility of coronaviruses [34].

The applied UV-C dosage (near 2 J/cm^2^) to each side of the mask is higher than the minimum recommended dosage by the literature [35], [36]. In this study, as was mentioned in other previously published [37], [38], [39] is necessary to emphasize that the diversity of N95 mask models makes it necessary to test every disinfection protocol with the available model of the mask. As a proof of concept in this work, N95 face mask model Serie 9510 by Nitta was used, a model approved by NIOSH (National Institute for Occupational Safety and Health, USA, approval number 84A-8118). The support for the mask was designed to avoid the shadow generation over the body of the mask from the support itself or from the straps. This is highlighted in the user manual, describing in detail how to locate the mask on the support. To guarantee a secure disinfection of the straps, a complement of their irradiation is suggested [40] using a disinfectant suitable for the material of the straps, it could be ethanol 70% or a quaternary amine depending on the mask model [4]. Always is necessary to avoid the direct contact of the disinfectant with the body of the mask.

The maximum number of processing times for the mask was not experimentally determined in our study. Although it could be done in the future, according to the literature is possible to estimate that with the applied UV-C dose the physical integrity of the mask is not significantly affected. Like other methods, before it makes to have a damaging effect, the physical integrity is compromised by the mechanical effect of donning and doffing of the masks [4]. According to this, and from a conservative perspective, we suggest three as the maximum number of processing times for using our method with each N95 mask [4], [5], [25]. Because repeated donning and doffing is an important cause of damage for the N95 masks, its extended use is prioritized before decontaminate and reuse them [41].

This work presents a device, which unlike others that have been designed this year [22], is accompanied by detailed instructions and plans for its construction and is released under a CC4 license. This allows easily adapt its use, by modifying the firmware it is possible to increase the applied dose or even the drawings provided allow structural modifications that could be made in the future. This protocol could be included in a two steps process, including a first step of decay of the viral load, storing the mask with controlled temperature and relative humidity conditions. This device was considered for disinfection mask contaminated with SARS-COV19. Other pathogens could be considered only after a specific study.

## 8 Human and animal rights

The work did not involve human or animal subjects.

## Declaration of Competing Interest

The authors declare that they have no known competing financial interests or personal relationships that could have appeared to influence the work reported in this paper.

## Figures and Tables

**Fig. A1:**
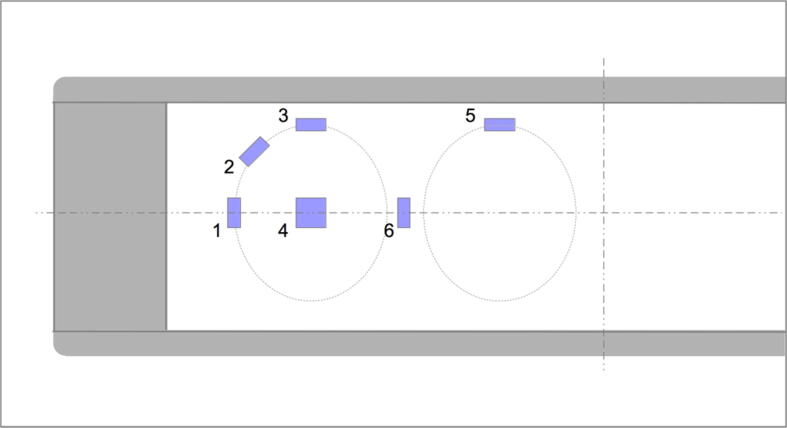
Scheme showing the masks’ support and the distribution of the points where the intensity measurements were done. The orientation of the detector is partially indicated in the figure.

**Table A1:** Measurements of intensity in the irradiation chamber at different points and for different surface orientations. The orientation is indicated by the angle of the surface to the vertical and by the letter e (intensity on the external surface of the mask) or i (intensity on the internal surface of the mask). The least irradiated points are highlighted in red.

## Appendix 1 LUCIA – UV-C light measurements

### Light intensity

We have carefully measured the light intensity in the irradiation chamber, in order to characterize their distribution over the surface of the masks. We have measured some characteristic points, that are distributed in only one quadrant assuming the symmetry of the irradiation chamber, as shown in Fig. A1. For each point, we have measured the intensity received on a surface having different orientations (different angles from the vertical position) and facing the radiation that arrives at the external and internal surface of the mask.

In Table A1 we show the intensity data measured with the UV-C germicidal light detector ZED SMART METER with a detector head D-SiCONORM SN:25437.

As the detector head used by this device is relatively big, we have also built a much smaller UV-C light probe using the UV-C sensor GUVC-S10GD from GENUV. This photodiode, that is specially designed for application using UV light in the C-band, have their response in spectrum band 220–280 nm. This home-built detector has been calibrated with the commercial detector. The electronic for this device is described in the corresponding supplemental material.

In Table A1, the position is indicated by a number that corresponds to the position shown in the figure. The orientation is shown in the figure and is also defined by the angle of the detector surface with the vertical direction (direction perpendicular to the plane of the masks' support). Moreover, a detector in any position can have two different orientations, either facing the light arriving at the external surface of the mask, or to the internal surface. This orientation is indicated in the table by the letter e: external or i: internal.

To guarantee that every point in the mask surface receives the minimum required UV-C energy dose, we have determined the exposure time considering the least irradiated points, i.e. positions and orientations 2,0,e and 3,0,e. As these orientations correspond to vertical surfaces, an orientation that we have never observed in any mask model, we have instead used the value of the intensity of 2.0 mW/cm^2^ corresponding to a realistic angle. We have fixed the exposure time of our equipment based on this value for the minimum intensity.

For example, for an energy dose of 1 J/cm^2^, the exposure time is 9 min. This exposure time takes into account the thermal transient observed in the intensity.

The intensities corresponding to the least irradiated points are dependent on the quality of the aluminum used to build the reflectors. We recommend carefully measure the UV-C intensity before fixing the exposure time of your equipment.

### Light spectrum

We have also measured the spectrum of the light source (30 Watt Philip discharge tubes model TUV T8 1 SL/25 UV), using a spectrometer Ocean Optics S2000. As can be seen (Fig. 5 of the article) in the germicidal spectral band (220 – 280 nm), the light source concentrates most of the emitted energy in the narrow spectral line of mercury (Hg) centered at approximately 254 nm. The emission in other Hg lines in this band is almost negligible.
